# Roles of BTLA in Immunity and Immune Disorders

**DOI:** 10.3389/fimmu.2021.654960

**Published:** 2021-03-29

**Authors:** Zhaochen Ning, Keyan Liu, Huabao Xiong

**Affiliations:** ^1^ Institute of Immunology and Molecular Medicine, Jining Medical University, Jining, China; ^2^ Jining Key Laboratory of Immunology, Jining Medical University, Jining, China; ^3^ Department of Public Health, Jining Medical University, Jining, China

**Keywords:** BTLA, coinhibition, inflammation, cancer immunotherapy, HVEM

## Abstract

B and T lymphocyte attenuator (BTLA) is one of the most important cosignaling molecules. It belongs to the CD28 superfamily and is similar to programmed cell death-1 (PD-1) and cytotoxic T lymphocyte associated antigen-4 (CTLA-4) in terms of its structure and function. BTLA can be detected in most lymphocytes and induces immunosuppression by inhibiting B and T cell activation and proliferation. The BTLA ligand, herpesvirus entry mediator (HVEM), does not belong to the classic B7 family. Instead, it is a member of the tumor necrosis factor receptor (TNFR) superfamily. The association of BTLA with HVEM directly bridges the CD28 and TNFR families and mediates broad and powerful immune effects. Recently, a large number of studies have found that BTLA participates in numerous physiopathological processes, such as tumor, inflammatory diseases, autoimmune diseases, infectious diseases, and transplantation rejection. Therefore, the present work aimed to review the existing knowledge about BTLA in immunity and summarize the diverse functions of BTLA in various immune disorders.

## Introduction

B and T lymphocyte attenuator (BTLA) is a member of the CD28 superfamily. Its gene is localized in the q13.2 region of chromosome 3 and consists of 5 exons with a total length of 870 bp. The protein structure of BTLA is similar to programmed cell death-1 (PD-1) and cytotoxic T lymphocyte associated antigen-4 (CTLA-4), which includes extracellular domain, transmembrane domain and cytoplasmic domain (1, 2). The cytoplasmic domain contains growth factor receptor-bound protein-2 (Grb-2) association motif, immunoreceptor tyrosine-based switch motif (ITSM), and immunoreceptor tyrosine-based inhibitory motif (ITIM). HVEM binding activates tyrosine phosphorylation of the ITIM in BTLA and leads to the recruitment of the Src homology domain 2 (SH2)-containing protein tyrosine phosphatases, SHP-1 and SHP-2, which generally mediate immunosuppressive effects (3, 4). Interestingly, binding of the Grb-2 association motif with Grb-2 leads to the recruitment of PI3K protein subunit p85 and T cell activation (5). At present, herpes virus entry mediator (HVEM), which belongs to the tumor necrosis factor receptor superfamily, is the only identified ligand that can be detected in human cells (1). HVEM can interact with BTLA in a *cis* or *trans* manner. On cells coexpressing BTLA and HVEM, BTLA can interact with HVEM in *cis*, whereas *trans* interaction occurs when BTLA and HVEM are expressed on different cells (2, 6). In addition to BTLA, HVEM can also interact with CD160, lymphotoxin-α, LIGHT (TNFSF14), and synaptic adhesion-like molecule 5 (SALM5) (7–9). Notably, BTLA and CD160 compete for the same binding site within the CRD1/CRD2 region of HVEM, while LIGHT independently binds the opposite side of HVEM within the CRD2/CRD3 region (1, 8, 10). BTLA can be extensively expressed in lymph nodes, thymus, and spleen, but little or no expression is detected in organs, such as the heart, kidney, brain, and liver (11). Among immune cells, BTLA is mainly expressed in B and T cells. In mouse spleen, the expression of BTLA is higher in B cells than T cells. With regard to T cells, BTLA expression can be detected on both CD4^+^ and CD8^+^ T cells, whereas CD4^+^ T cells express more BTLA than CD8^+^ T cells (12). Besides, its expression can also be detected in innate immune cells, such as dendritic cells (DCs) and monocytes (13). HVEM binding to BTLA exerts direct negative effects on the proliferation and activation of B and T cells (14). As a result, they affect a variety of physiopathological processes. This review aimed to summarize the recent results on the effects of BTLA in regulating immune cell function and to explore its potential role in immune disorders.

## BTLA Effects in Immunocytes

### BTLA and T Cells

BTLA levels vary during T cell differentiation and activation processes. Specifically, BTLA is expressed in naive T cells, and the expression transiently increases upon activation, but is decreased in activated T cells (15–18). Moreover, the interaction manners between BTLA and HVEM also changes. Under homeostatic conditions, HVEM and BTLA interact in *cis* to provide intrinsic inhibitory signals. Upon T cell activation, HVEM is internalized, which allows BTLA to interact with HVEM in *trans* (6, 10). BTLA-deficient mice exhibit no defects in lymphocyte development, suggesting that BTLA is not necessary for T cell development (19). However, BTLA can significantly inhibit T cell activation and proliferation. BTLA-deficient T cells show increased proliferation (4) and are hyperresponsive to TCR-mediated activation (19). BTLA agonistic monoclonal antibody (mAb) suppresses the proliferation of T cells and the secretion of interleukin (IL)-10 and interferon γ (IFN-γ) upon anti-CD3 stimulation (20). Antibodies targeting BTLA enhance the proliferation of CD8^+^ T cells and blocking BTLA in combination with PD-1 is found to be effective in enhancing the exhausted human T cell response (21). Krieg et al. found that BTLA^-/-^ mice showed an elevated memory CD8^+^ T cell count and proposed that the increased T cell proliferation mediated by BTLA deficiency was associated with alterations in T cell memory subsets but not co-inhibition (22). However, Deppong et al. discovered that T cells with BTLA deficiency exhibited no enhanced proliferation, but showed decreased death (23). In addition, BTLA contributes to the induction of peripheral tolerance in CD4^+^ and CD8^+^ T cells (24). In naive T-cells, BTLA and HVEM form a *cis*-heterodimeric complex, which blocks other co-signaling molecules from binding to HVEM and stimulating the NF-κB signaling pathway, thus maintaining the tolerance of T cells (6). BTLA engagement has been suggested to facilitate SHP-1 and SHP-2 recruitment (3, 4). However, Chemnitz et al. reported that SHP-1 recruitment was not related to the function of BTLA, as blocking SHP-1 recruitment did not affect the function of BTLA (25). Paradoxically, it was recently shown that BTLA preferentially recruits SHP-1 to suppress T cell signaling more efficiently (26, 27). γδT cells can quickly produce inflammatory cytokines at sites of barrier to protect against pathogens. BTLA can limit γδ T cell numbers and control proliferation and cytokine secretion in mature lymph node γδ T cells (28). Vγ9Vδ2 cells are the main subset of γδ T cells in peripheral blood that are reactive to tumors and microbial agents, and BTLA was found to be strongly expressed in resting Vγ9Vδ2 T cells. BTLA engagement inhibited Vγ9Vδ2 T cell proliferation, while targeting BTLA resulted in the enhancement of Vγ9Vδ2 TCR-mediated signaling (29).

### BTLA and B Cells

BTLA in B cells has rarely been studied compared to that in T cells. In human B cell subsets, mature peripheral B cells display the most significant BTLA level, whereas a uniform BTLA expression is observed in naive, transitional, and memory peripheral B cells, and the lowest level is detected in bone marrow-derived precursor B cells (30). In addition, BTLA levels are downregulated in B cells of the aged, which is related to the decreased reactivity to the trivalent influenza vaccine (31). In mouse spleen, compared to T cells, B cells display higher surface BTLA level (12, 32). Consistent with its effect on the development of T cells, BTLA does not significantly affect the development of B cells, because normal growth of lymphocytes is detected in mice with BTLA deficiency (4, 19). Moreover, it was found that BTLA/HVEM ligation can suppress the functions of B cells (such as proliferation, cytokine secretion, and co-stimulatory molecule upregulation) (30, 33). Engagement of BTLA recruits SHP-1, leading to reduced activation of BCR downstream signaling molecules (30). However, Zhang et al. reported that BTLA antibodies had no effect on lipopolysaccharide (LPS)- or anti-IgM antibody-induced B cell proliferation *in vitro* (34). Regarding helper T (Th) cells, follicular Th (Tfh) cells represent the dominant subset that promotes antibody secretion by B cells. Mintz et al. discovered that HVEM engagement of BTLA on Tfh cells reduced T cell receptor (TCR) signaling and CD40 ligand mobilization to the synapse, thereby reducing the help to B cells and inhibiting B cell proliferation (35). Additionally, it was found that BTLA could inhibit IL-21 production by Tfh cells to suppress the development of germinal center B cells and subsequent IgG responses (36).

### BTLA and DCs

As its name implies, BTLA shows preferential expression in T and B cells, yet its expression can also be detected in additional immune cells, such as DCs. Immature DCs express lower levels of BTLA, and BTLA expression increases with maturation (37). The number of DCs in BTLA^-/-^ mice spleen was found to be similar to that in wild-type mice, suggesting that BTLA is not essential for DC development (38). The HVEM-BTLA pathway plays an important role in regulating DC homeostasis. The lymphotoxin β receptor signaling pathway triggers the proliferation of DCs, while the HVEM-BTLA signaling pathway suppresses DC proliferation, suggesting that the HVEM-BTLA pathway provides an inhibitory checkpoint for DC homeostasis (39). Xin et al. reported that BTLA overexpression suppressed DC maturation and enhanced the immune tolerance of immature DCs (40). BTLA can inhibit toll-like receptor 4 signaling and proinflammatory cytokine production in DCs, thereby inhibiting LPS-induced endotoxic shock (38). Jones et al. discovered that BTLA^+^ DCs governed the conversion of peripheral Treg cells by upregulating CD5, thus enhancing the tolerance of peripheral Treg cells (41). Moreover, active pulmonary tuberculosis drives BTLA expression in DCs, thereby inhibiting Th17 and Th22 responses induced by DCs and promoting Treg and Th2 differentiation (42). Further, urothelial cancer induces the overexpression of BTLA in DCs, resulting in reduced secretion of effector cytokines (43).

## The Functions of BTLA in Immune-Related Diseases

### BTLA and Tumors

BTLA is found to be expressed in tumor-infiltrating lymphocytes (TILs) and is often associated with impaired anti-tumor immune response. Upregulated BTLA expression in gallbladder cancer (GBC) plays a role in inhibiting anticancer immunity, and an increased proportion of BTLA^+^CD8^+^ cells is related to the unfavorable outcome of GBC patients (44). In hepatocellular carcinoma (HCC) cases, the expression level of BTLA markedly increases in circulating CD4^+^ cells rather than in CD8^+^ T cells (45, 46). Besides, blocking the BTLA/HVEM signaling pathway can promote IFN-γ secretion by circulating CD4^+^ and CD8^+^ T cells (45). BTLA^+^ T cells show increased levels of additional checkpoint molecules, such as PD-1, lymphocyte-activation gene-3, T cell immunoglobulin and mucin-do-main-containing molecule-3 (TIM-3). In addition, these cells exhibit a poorly differentiated phenotype, reduced cytolysis, and increased proliferation potential among patients with diffuse large B-cell lymphoma (DLBCL). Further, increased BTLA levels are related to an advanced disease stage in DLBCL patients (47). BTLA expression is elevated in T cells from patients with melanoma (48, 49). Furthermore, PD-1^+^TIM-3^-^CD8^+^ T cells expressing BTLA are the largest tumor-specific CD8^+^ T cell subset among melanoma cases that show partial dysfunction with decreased IFN-γ production compared with BTLA^-^ T cells (49). BTLA is highly expressed in type I NKT cells in murine autochthonous mammary tumors, and BTLA-neutralizing antibodies can inhibit tumor proliferation and pulmonary metastasis (50). BTLA expression was also found to be upregulated in T cells from patients with lung cancer (51). In addition, soluble BTLA (sBTLA) in plasma is significantly associated with the risk of death in patients with clear cell renal cell cancer, suggesting that sBTLA has a similar role to membranous BTLA in inhibiting T cell response (52). However, some studies have reported contradictory results. For instance, as suggested by Song et al., BTLA levels decreased in colorectal cancer tissues in comparison with levels in matched non-carcinoma tissues. In addition, decreased BTLA levels predicted poor overall survival and less LNM (53). Notably, BTLA is suggested to trigger both inhibitory and survival signaling, and may have a context-specific function in TILs (7). Although BTLA is a co-inhibitory receptor, higher frequencies and numbers of BTLA-expressing CD8^+^ TILs are markedly associated with positive adoptive cell therapy responses among melanoma cases (54). This team further found that BTLA marks a less-differentiated TIL subset, which displays enhanced resistance to apoptosis and improved survival after tumor killing (55, 56). Besides, growing evidence suggests that polymorphisms of BTLA increase susceptibility to a wide range of cancers. Rs1982809 is a functional single nucleotide polymorphism (SNP) that affects BTLA 3′-UTR activity and BTLA expression, and has been found to be associated with a higher incidence of renal cell carcinoma (57), chronic lymphocytic leukemia (58) and esophagogastric junction adenocarcinoma (59). Five BTLA gene SNPs were found to be related to progesterone receptor, estrogen receptor, tumor size, p53, and C-erbB-2 statuses among Chinese females from the northeast Heilongjiang Province (60). However, Cao et al. found no significant relationship between BTLA polymorphisms and esophageal squamous cell carcinoma (61).

### BTLA in Sepsis and Inflammatory Diseases

BTLA negatively regulates T cell-mediated inflammation. BTLA^-/-^ mice experience a prolonged duration of allergic airway inflammation and enhanced recruitment of inflammatory cells relative to those in wild-type mice (62). Moreover, DNFB-mediated contact hypersensitivity, CD8^+^ T cell proliferation, and IFN-γ generation increased in BTLA^-/-^ mice, whereas these effects were reversed by an agonistic anti-BTLA antibody (63). The BTLA^+^ T cell frequency is elevated in the mucosa and blood of mice with colitis, and BTLA can control inflammatory responses by upregulating Foxp3 expression (64). In a Con A challenge model of acute hepatitis, BTLA^-/-^ mice showed reduced survival and increased early secretion of serum cytokines, which was partly due to the negative regulation of NKT cells by BTLA (65). Moreover, Shi et al. discovered that BTLA could induce the self-tolerance of CD8^+^BTLA^+^ T cells to reduce the attack on hepatocytes, thus regulating hepatic homeostasis in a Con A-induced hepatitis model in zebrafish (66).

Notably, a growing body of research suggests that BTLA plays an important role in sepsis. Sepsis mortality is prevented in mice with BTLA deficiency, which presents with reduced leukocyte recruitment into the peritoneum, reduced IL-10 secretion, increased bacterial clearance, and suppressed neutrophil activation (67). Besides, it is further found that critically ill sepsis patients show a markedly increased proportion of BTLA^+^ CD4^+^ lymphocytes in peripheral blood, which is associated with a subsequent infection (68). The BTLA level in Tregs (69) and plasma concentration of sBTLA (70, 71) are elevated in sepsis cases, and they are related to sepsis severity. However, some studies have reported different results. Specifically, relative to that in normal subjects, the number of BTLA^+^CD4^+^ T cells decreased among patients with severe sepsis, and a lower percentage of BTLA^+^CD4^+^ T cells during the early stage of sepsis was associated with the severity and mortality of sepsis patients (72). Furthermore, Spec et al. reported that differences in BTLA levels in CD4^+^ and CD8^+^ T cells were not statistically significant between patients with *Candida* sepsis and controls (73). Interestingly, different clones of BTLA antibodies have two contradictory effects, including neutralizing and potentiating effects on BTLA-mediated signaling (74, 75). Cheng et al. reported that BTLA antibody treatment increased the levels of cytokines and chemokines and promoted the recruitment of inflammatory cells into the peritoneal cavity, leading to deteriorated organ damage and increased mortality in mice with hemorrhagic shock/sepsis (76). However, Kobayashi et al. found that a BTLA antibody rescued mice from LPS-induced endotoxic shock (38). The authors also suggested that mice with BTLA deficiency showed high susceptibility to LPS-induced endotoxic shock and that LPS-mediated production of IL-12 and tumor necrosis factor α significantly increased in macrophages and DCs of mice with BTLA deficiency (38). These discrepant results from different studies may be related to the different functions of the antibodies used. Moreover, genetic variations in BTLA are associated with sepsis morbidity. In addition to increasing cancer susceptibility, Rs1982809 is also associated with the incidence of sepsis and multiple organ dysfunction scores (77).

### BTLA and Autoimmune Diseases

BTLA is suggested to play a vital role in the protection from autoimmunity. Mice with BTLA deficiency exhibit increased antigen-specific IgG responses and are sensitive to experimental autoimmune encephalomyelitis (EAE) (4, 78). BTLA deficiency results in spontaneous development of autoimmune hepatitis-like disease, which is characterized by elevated transaminase levels, interface hepatitis, and hepatic spotty necrosis (79). The number of BTLA-expressing B cells and CD19^+^/BTLA^+^/IL-10^+^ cells obviously decreased in multiple sclerosis (MS) cases, while the remission of fingolimod-induced relapsed-remitted MS is related to a significantly increased number of CD19^+^/BTLA^+^/IL-10^+^ B lymphocytes (80). In patients with active systemic lupus erythematosus (SLE), the proportion of CD4^+^BTLA^+^ T cells is markedly decreased (81). Sawaf et al. found that in SLE cases, BTLA had a lower ability to suppress the activation of T cells, and such impaired BTLA function in lupus CD4^+^ T cells was found to be related to disease activity (82). BTLA deficiency accelerates the lupus-like phenotype in autoimmune-prone MRL-lpr/lpr mice, and severe lymphocytic infiltration is detected in the lungs, kidneys, salivary glands, joints, and pancreas (83). However, a study conducted in the Japanese population showed that there was no significant difference in haplotypes, genotypes, and alleles of the BTLA gene between SLE and healthy groups (84). Anti-BTLA mAb treatment can delay the onset of autoimmune diabetes in non-obese diabetic (NOD) mice, increase the proportion of Tregs, and direct the cytokine milieu away from autoimmunity (85). Transferring transgenic BTLA-expressing DCs into NOD mice markedly triggers the tolerance of CD8^+^ T cells, while attenuating the autoimmune diabetes severity (86). In rheumatoid arthritis (RA) patients, BTLA-expressing CD3^+^/CD4^+^/CD8^+^ T cell proportions are remarkably increased, and the swollen joint count is negatively correlated with the percentage of BTLA^+^CD8^+^ T cells (87). Lin et al. found that the C+800T SNP in the BTLA gene was associated with RA susceptibility (88). Besides, the 590C SNP, a functional polymorphism of the BTLA gene, is markedly associated with RA susceptibility in the Japanese population (89).

### BTLA and Infectious Diseases

Since BTLA can provide both inhibitory and pro-survival signals to T cells, it plays a dual role during infection. BTLA expression increases in various infectious diseases, which is generally related to an impaired immune response against infection. BTLA is strongly upregulated in both CD4^+^ and CD8^+^ T cells from COVID-19 patients (90). Similarly, BTLA levels increase in CD4^+^ and CD8^+^ T cells from patients with pulmonary tuberculosis, and this is related to disease progression (91). During infection with primary cytomegalovirus (CMV), BTLA is highly induced in CD8^+^ T cells, and BTLA blockade enhances CMV-specific CD8^+^ T cell proliferation (92). Increased BTLA level in intrahepatic lymphocytes is related to impaired T cell responses in the process of chronic hepatitis B virus (HBV) infection, whereas blocking BTLA promotes cytokine production and T cell proliferation (93). In patients with chronic HBV infection, a subset of antigen-specific CD8^+^ T cells with low IFN-γ production express high levels of BTLA, and these cells play a vital role in the regulation of CD8^+^ T cell responses *via* BTLA signaling (94). In patients with severe community-acquired pneumonia and in mice with acute lung inflammation, circulating BTLA^+^CD4^+^ lymphocyte proportions markedly increased, whereas agonistic anti-BTLA antibody reduced the activation of the NF-κB pathway and attenuated the inflammatory responses (95). In mice infected with the parasitic nematode *Strongyloides ratti*, BTLA expression in CD4^+^ T cells was upregulated. Additionally, deficiency of either BTLA or HVEM leads to decreased amounts of adult parasites in the small intestine, while reducing the larval output in the process of infection (96). Moreover, BTLA^-/-^ mice have also shown resistance to infection in several other disease models. During infection with murine hepatitis virus strain-3 (MHV-3), BTLA^-/-^ mice show markedly improved spleen and liver injuries and reduced mortality (97). BTLA suppresses both the innate and T/B cell-dependent adaptive immune responses during experimental malaria, whereas BTLA^-/-^ mice show markedly decreased parasitemia, and early clearance of infections (98). Furthermore, BTLA^-/-^ mice are more resistant to listeriosis infection (99), whereas targeting BTLA promotes primary and memory T cell responses (100) and accelerates early bacterial clearance (99). Interestingly, BTLA^-/-^ mice have a decreased number of *Listeria*-specific CD8^+^ T cells, which reveals the dual effects of BTLA in regulating anti-intracellular pathogen responses in the host (100, 101). Furthermore, HVEM expression in CD8^+^ T lymphocytes and BTLA expression in other cell types are necessary for the optimal survival of effector and memory T cells, suggesting that the BTLA/HVEM pathway enhances activated CD8^+^ T cell survival in the process of *Listeria* infection (101). During vaccinia virus infection, BTLA or HVEM deficiency markedly damages the survival of effector CD8^+^ T cells and protective immune memory (102). BTLA^+^ αβ T cells display a central memory phenotype to combat *Mycobacterium tuberculosis* (Mtb) infection, as manifested by increased cell proliferation and cytokine secretion, suggesting that BTLA expression in αβT cells is involved in protective immune memory against Mtb infection among patients with active pulmonary tuberculosis (103).

### BTLA and Transplantation Rejection

The inhibitory role of BTLA on T cells has also been supported by transplantation studies. BTLA can inhibit donor anti-host T cell responses and ameliorate graft-versus-host disease (GVHD) resulting from allogeneic bone marrow transplantation (104, 105). In patients with acute renal allograft rejection, BTLA levels in peripheral CD3^+^ T lymphocytes are markedly lower than those in normal controls who show stable functions of the transplanted kidney (106, 107). Agonistic anti-BTLA antibody treatment prolongs cardiac allograft survival in mice with increased CD4^+^CD25^+^Foxp3^+^ cells and reduced IL-2 and IFN-γ production (108). Furthermore, BTLA overexpression in a rat model can markedly suppress acute kidney rejection and extend allograft survival (106). Partially MHC-mismatched allografts can potently induce BTLA expression, while targeting BTLA prompts rapid rejection (109). Agonistic anti­BTLA antibody combined with CTLA-4 immunoglobulin treatment can prolong the survival of islet allografts, while promoting indefinite graft acceptance (110, 111). Moreover, according to the latest research, overexpression of BTLA combined with CTLA-4 in kidney transplant recipients reduced IL-2 production, suppressed T cell proliferation, improved graft function, attenuated acute T cell-mediated rejection, and extended graft survival (112). However, there have been some contradictory reports. For example, Del Rio et al. reported that BTLA blockade alleviated acute GVHD reaction in an F1 transfer semiallogeneic murine model (113, 114). Rodriguez-Barbosa et al. found that targeting BTLA/HVEM showed no effect on modulating graft rejection across a fully MHC mismatched barrier and that the donor-specific allogeneic immune response was independent of the HVEM/BTLA signaling pathway (115). Besides, Wang et al. explored the role of BTLA and HVEM polymorphisms in antibody-mediated rejection in renal transplant recipients and found that none of the polymorphisms identified was associated with antibody-mediated rejection (116).

## Perspectives

BTLA has been found to affect the function of a variety of immune cells and plays an important role in many immune-related diseases (**Table 1**). There is growing evidence that BTLA can provide both inhibitory and pro-survival signals to T cells. As a result, BTLA is found to play dual roles in tumor (7, 55, 56) and infection (101, 102) immunity, and the function of BTLA in related diseases may be context specific. BTLA can be expressed by numerous cell types. At present, the existing studies on BTLA mainly focus on T and B cells, and thus studies on the function of BTLA in other immune cells should be strengthened. Numerous clones of anti-BTLA antibodies have been developed, and the main antibody clones such as 6A6 and 6F7 were summarized in the review by Crawford and Wherry (75). Studies have examined the role of anti-BTLA antibodies in tumor, inflammatory diseases, and transplantation rejection. The findings show that BTLA-neutralizing antibodies can inhibit tumor development (50), while agonistic anti-BTLA antibody treatment reduces inflammation (63, 95) and prolongs allograft survival (108, 110, 111). However, these antibodies may not always act exactly as predicted, since different BTLA antibodies may have opposite functions. Confusingly, even the same clone antibody has been reported to exert opposite effects (75, 85, 117). Furthermore, considering that BTLA/HVEM can deliver bidirectional signals, antibodies targeting BTLA will cause complex biological effects. Although few therapeutic agents targeting inhibitory receptors (such as CTLA-4Ig and anti-PD-1) are being used in clinical trials, the complexity of BTLA signaling system remains a challenge for the development of targeted therapies.

**Table 1:** Reported functions of BTLA in immune-related diseases.

Disease	Reported functions (references)
Tumors	◾ BTLA expression increases in patients with GBC (44), HCC (45, 46), DLBCL (47), and melanoma (48, 49), and is associated with poor prognosis.
Inflammatory diseases	◾ Alleviates allergic airway inflammation (62), contact hypersensitivity (63), colitis (64), and acute hepatitis (65) in mice.
Sepsis	◾ Increased BTLA correlates with sepsis severity in patients (68–71). ◾ BTLA^-/-^ mice show reduced mortality (67).
Autoimmune diseases	◾ The expression of BTLA is decreased in MS (80) and SLE (81) patients, and increased in RA (87) patients. ◾ Decreases susceptibility to EAE (4, 78), autoimmune hepatitis (79), and autoimmune diabetes (85, 86) in mice.
Infectious diseases	◾ BTLA expression increases in patients with COVID-19 (90), Mtb (91), CMV (92), and HBV (93) infection. ◾ BTLA^-/-^ mice are more resistant to helminth (96), MHV-3 (97), *Plasmodium yoelii* strain 17NL (98), and listeriosis infection (99).
Transplantation rejection	◾ Improves GVHD (104, 105) and prolongs allograft survival in animal models (106, 108–112).

**Figure 1:**
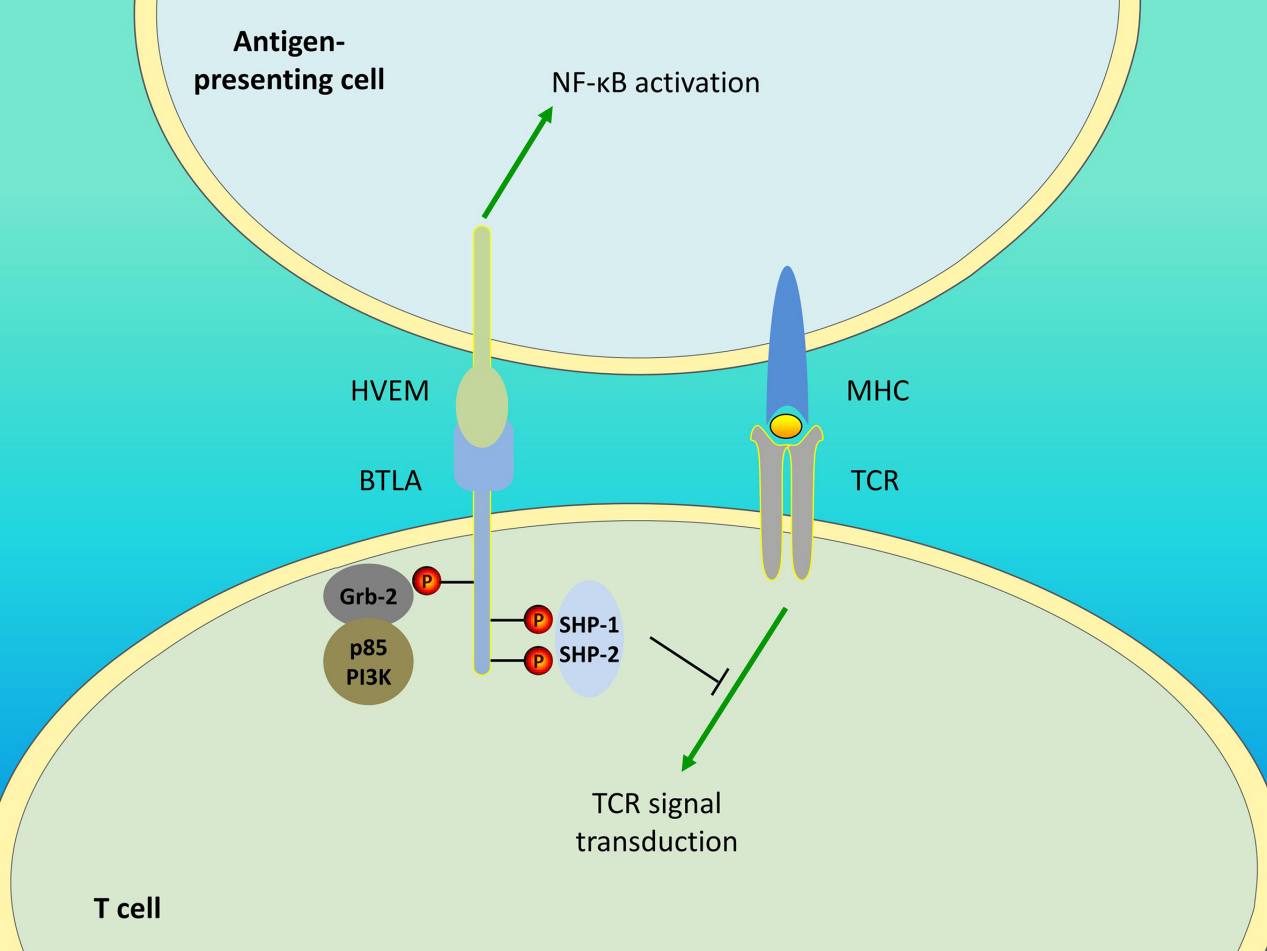
Bidirectional signaling between BTLA and HVEM. Engagement of BTLA leads to SHP-1 and SHP-2 recruitment in T cells, thereby downregulating TCR signaling and delivering inhibitory signals. The Grb-2 association motif binds to Grb-2, leading to the recruitment of PI3K protein subunit p85 and the stimulation of the PI3K signaling pathway. Additionally, BTLA/HVEM signaling is bidirectional. BTLA/HVEM engagement induces HVEM-mediated NF-κB activation in antigen-presenting cells, thus providing proinflammatory and pro-survival signals.

In addition to BTLA, HVEM interacts with LIGHT and CD160. All the four molecules form the HVEM/BTLA/CD160/LIGHT signaling network. For HVEM, binding to BTLA mediates the inhibitory effect, while binding to LIGHT mediates the activation effect. The role of CD160 remains controversial as it generates activating as well as inhibitory signals. In response to HVEM ligation, CD160 delivers coinhibitory signals to CD4^+^ T cells (8), whereas it provides costimulatory signals to CD8^+^ T (118) and NK (119) cells. Additionally, BTLA can provide negative feedback for NK cell activation by competing with CD160 (119). Therefore, HVEM is a bidirectional switch, and its outcome is determined by the ligand involved. In addition, BTLA/HVEM signaling can antagonize LIGHT/HVEM signaling-mediated effector cell activation (14) and additional studies are needed to elucidate which is the predominant pathway. Furthermore, BTLA/HVEM signaling is also bidirectional. Although BTLA delivers inhibitory signals, its engagement with HVEM induces HVEM-mediated NF-κB activation, which is important for the induction of proinflammatory and cell survival genes (120). This also suggests the possibility that the pro-survival effect mediated by BTLA/HVEM ligation may be caused by HVEM-NF-κB signaling rather than BTLA signaling. The unique interaction between BTLA and HVEM allows for a system of bidirectional signaling (**Figure 1**), which contributes to the understanding of the opposite or dual roles of HVEM and BTLA and the approach for their specific targeting in treatment.

## Author Contributions

ZN and KL are responsible for preparation of the published work. HX is responsible for supervision, review and editing and funding acquisition. All authors contributed to the article and approved the submitted version.

## Funding

The present study was funded by the National Natural Science Foundation of China (No. 82003027), Doctoral Startup Fund of Jining Medical University (NO. 2017JYQD24), Research Fund for Academician Lin He New Medicine (NO. JYHL2018MS11), and Chinese and foreign cooperation cultivation project of Jining Medical University.

## Conflict of Interest

The authors declare that the research was conducted in the absence of any commercial or financial relationships that could be construed as a potential conflict of interest.
